# Early-Onset Atopic Dermatitis in Children: Which Are the Phenotypes at Risk of Asthma? Results from the ORCA Cohort

**DOI:** 10.1371/journal.pone.0131369

**Published:** 2015-06-24

**Authors:** Flore Amat, Philippe Saint-Pierre, Emmanuelle Bourrat, Ariane Nemni, Rémy Couderc, Emmanuelle Boutmy-Deslandes, Fatiha Sahraoui, Isabelle Pansé, Martine Bagot, Sébastien Foueré, Jocelyne Just

**Affiliations:** 1 Department of Allergology—Centre de l’Asthme et des Allergies, Hôpital d’Enfants Armand Trousseau, Assistance Publique-Hôpitaux de Paris, Paris, France; 2 Equipe EPAR, Institut Pierre Louis d’Epidémiologie et de Santé Publique, UMR_S1136, INSERM, Paris, France; 3 UPMC Univ Paris 06, Sorbonne Universités, Paris, France; 4 Laboratory of Theoretical and Applied Statistics-Laboratoire de Statistiques Théoriques et Appliquées, UPMC Univ Paris 06, Sorbonne Universités, Paris, France; 5 Department of Dermatology, Hôpital Saint-Louis, Assistance Publique-Hôpitaux de Paris, Paris, France; 6 Laboratory of Biochemistry and Molecular Biology- Laboratoire de Biochimie et Biologie Moléculaire, Hôpital d’Enfants Armand Trousseau, Assistance Publique-Hôpitaux de Paris, Paris, France; 7 Department of Biostatistics and Medical Informatics-Service de Biostatistiques et informatique médicale, Hôpital Saint-Louis, Assistance Publique-Hôpitaux de Paris, Paris, France; 8 U717, INSERM, Université Paris 7, Paris, France; Université Paris Descartes, FRANCE

## Abstract

**Background:**

Atopic dermatitis (AD) is known to predate asthma and other atopic disorders described under the term “atopic march”. However, this classic sequence is not always present and only a few studies have addressed children at risk of developing asthma. The objective of this study is to define early-onset AD phenotypes leading to asthma.

**Methods:**

We performed a cluster analysis with 9 variables of 214 infants with early-onset AD prospectively enrolled in the ORCA cohort and followed each year on the occurrence of asthma until the age of 6.

**Results:**

We identified 3 clusters - **cluster 1** (n = 94) with low to no sensitization to food (27.7%) or aeroallergens (10.6%) and moderate AD severity (SCORAD 25.29 +/- 14.6) called “AD with low sensitization”; - **cluster 2** (n = 84) characterized by a higher AD severity (SCORAD 32.66+/-16.6) and frequent sensitization to food (98.9%) or aeroallergens (26.2%), most likely multiple (96.4% for food allergens), called “AD with multiple sensitizations” - **cluster 3** (n = 36) with parental history, moderate AD severity (SCORAD 24.46+/-15.7), moderate rate of sensitization to food allergens (38.9%) (exclusively single) with no sensitization to aeroallergens, called “AD with familial history of asthma”. Percentages of children suffering from asthma at the age of 6 were higher in clusters 2 and 3 (36.1% and 33.3% respectively versus 14.9% in cluster 1, p<0.01).

**Conclusion:**

Two phenotypes in infants with early-onset AD convey a higher risk of developing asthma during childhood: multiple sensitization and familial history of asthma.

## Introduction

Atopic dermatitis (AD) has become a significant public health problem because of its increasing prevalence [1]. The relationship between AD and sensitization to aeroallergens has been previously described [2]. This progression from AD to sensitization to aeroallergens and then asthma may be defined as the natural history of atopic manifestations, described by the term “atopic march”. Atopic march is characterized by a sequence of atopic diseases in childhood, typically with AD predating the development of other allergic disorders later in life. It has been estimated that one-third to half of patients with AD will develop asthma [3]. However, debate continues as to whether this represents a causal relationship as the atopic march does not always follow this classic sequence. Although AD seems to be the first step leading to asthma especially when severe [4, 5] or early-onset [6], most studies performed on atopic march mechanisms have focused on birth cohorts or in the general population where this phenotype is quite rare [7, 8].

In this context, we set out to define phenotypes of early-onset AD leading to asthma in a prospective study using an unsupervised statistical approach. Because the mechanisms of progression from AD to asthma seem to be a combination of shared environmental factors and genetic background [9], analysis included environmental factors, familial history and biological markers of atopy.

## Patients and Methods

### Design and inclusion criteria

Patients were part of the ten-year (2002–2012) Observatory of Respiratory risks linked with Cutaneous Atopy (ORCA) study, resulting from the collaboration between two tertiary care centers, the Allergology Department at the Armand Trousseau Children’s Hospital and the Dermatology Department at the Saint-Louis Hospital, both in Paris, France. The study prospectively included children with early-onset AD living in Paris or its suburbs and referred to the Saint-Louis Hospital by a primary care physician. All the children meeting the following criteria were considered for inclusion: i. aged younger than 12 months, ii. with an active AD diagnosed by a dermatologist according to the United Kingdom Working Party criteria (UKWP) [10] and ISAAC questionnaire [11], iii. without any history of wheezing before the time of inclusion. It was offered to parents who entered the study to follow their child with a systematical reevaluation on atopic dermatitis, allergic and asthma status annually until the age of 6 years.

### Ethics

Both parents of each child provided written informed consent at inclusion. The study was specifically approved by the Institutional Review Board of the Medical Ethics Committee on Research of the Saint-Louis Hospital (Comité Consultatif de Protection des Personnes dans la Recherche Biomédicale, Hôpital Saint Louis, Paris France) in September 2001 under the ID number 2001/42. Data were collected for the study with respect to the confidentiality of patient records.

### Data collection at inclusion

Clinical data collected by a trained study binomial, including a dermatologist and an allergo-pulmonologist,were:

1. Age and gender.
2. Ethnic background (categorized as of Western Europe descent, African-Caribbean descent and other descent) and socio-economic status based on the highest level of occupation of the parents, categorized as low (low-level white-collar workers, blue-collar workers, and the unemployed), intermediate (intermediate white-collar workers, craftsmen, and shopkeepers) or high (high-level white-collar workers) [12].
3. AD severity was assessed by the auto-administered and physician-supervised objective SCORAD questionnaire [13]. We considered severity as mild when the SCORAD was under 15, moderate when the SCORAD was between 15 and 40 as high when the SCORAD was above 40 [14].
4. Food allergy was defined by relevant allergic symptoms (such as urticaria, difficulties to breathe, gastro-intestinal disorders), assessed by a structured questionnaire, following consumption of a food allergen and associated with an IgE sensitization to the same allergen, and was confirmed by the allergo-pulmonologist. Cow’s milk allergy was analyzed separately from other food allergies due to its particular good prognosis during childhood [15].
5. Finally, parental history of asthma.

Environmental factors were evaluated by questionnaire and included potential sources of biologic allergens in the household environment: presence of molds (reported visible molds, moldy smell, or both), furred pets (cats, dogs, and rodents) or cockroaches in the home [12]. Behavioral factors assessed included postnatal exposure to tobacco smoke (based on smokers in the home including mother, father, or other adult household members) and day care attendance during the first year of life [12].

Biological markers of atopy were measured in peripheral blood. These included IgEs for specific aeroallergens and food allergens (ImmunoCAP Phadiatop Infant; Phadia AB-Thermo Fisher Scientific; Uppsala, Sweden), less-specific markers such as blood eosinophilia (cell counting by automated Sysmex; Roissy, France) and total IgE (measured by ImmunoCAP Total IgE;; Phadia AB-Thermo Fisher Scientific; Uppsala, Sweden).

Thresholds were used to define increased levels, according to previous studies: increased blood eosinophilia was defined as a concentration of 470 eosinophils/mm^3^ or more and increased total IgE as a concentration of 45 kU/L or more [2, 16].

Sensitization was defined as a specific IgE concentration ≥0.35 kU/L in serum against one of the following aeroallergens and food allergens: house dust mite (HDM), cat and dog dander, pollens (birch tree, timothy grass, mugwort), cockroaches, cow's milk, hen’s egg, peanut, soy, fish and wheat.

Multiple sensitizations were defined as at least two positive specific IgEs to allergens [2].

### Prospective data collection

Children were followed-up annually both by a dermatologist and an allergo-pulmonologist annually from the age of 6 months until the age of 6 years.

Parameters recorded at each visit were:

1. Active AD during the year, assessed by the dermatologist using SCORAD and topical corticosteroid use. Remission of AD was defined as the absence of active AD during the year before the visit (SCORAD equal to zero and absence of topical corticosteroid use)
2. Occurrence of food allergy as defined at the time of inclusion, confirmed by the allergo-pulmonologist
3. Biological markers of atopy (i.e. IgEs for specific aeroallergens and food allergens, total IgE and blood eosinophilia, using the same thresholds as at the time of inclusion)
4. Respiratory outcome defined according to parents’ answers to ISAAC questionnaire administered during the visit [11] and collected by the allergo-pulmonologist. Briefly, diagnosis of asthma was considered if parents answered “yes” to one or more of the following questions: “has your child ever had a medical diagnosis of asthma?”, “in the last 12 months, has your child’s chest sounded wheezy during or after exercise?”, “in the last 12 months, has your child had a dry cough at night, apart from a cough associated with a cold or a chest infection? “; or if parents answered “three times or more” to the question “has your child ever had wheezing or whistling in the chest at any time in the past?”[17]. Allergic asthma was defined by symptoms of asthma associated with an IgE sensitization to one or more aeroallergens.

Dataset about the cohort is avalaible in the Supporting Information file (S1 File)

### Statistics

According to the prevalence of asthma in 6-year-old children with AD [3] and studied risk factors, it was estimated that 246 subjects would be required to show a significant difference of 0.05 between groups.

#### Variable reduction and data transformation

The initial database included 36 variables. A reduction was required to perform the cluster analysis. Variables that were clinically redundant were excluded or reduced to avoid bias in the cluster analysis (such as topical corticosteroid use and SCORAD to evaluate disease severity, percentage along with absolute number of eosinophils, or multiple sensitizations to allergens along with sensitization to allergens taken individually). Environmental parameters were not included in the cluster analysis but were crossed with the final clusters. Subjects were required to have a full data set of the variables used in the cluster analysis: demographic data, variables previously reported to affect disease severity, biological markers of atopy

#### Statistical analysis

Statistical analysis was performed with R version 2.12.0 (http://www.r-project.org). A hierarchical bottom-up clustering method was used to perform an unsupervised classification of the population [18]. This agglomerative approach begins with each subject as a separate cluster and merges them into successively larger clusters. Ward’s linkage (sample merged into larger clusters to minimize the within-cluster sum of squares) and the Euclidian distance were considered (R package, cluster). Before hierarchical clustering analysis, a dissimilarity matrix needs to be evaluated. All variables are standardized to give them the same weight in the analysis. Gower’s standardization was applied for this [19]. The dendrogram provided by the method helps to identify the number of clusters since it gives information on the cost of merging clusters. A classification into 2 groups was suggested but discarded because it only reflected one dimension, the AD outcome. No clear indication was given for the choice between 3, 4 or 5 groups. However, we discarded classification into 4 or 5 groups because the subgroups were too small and the interpretation complex and lacking in clarity. Finally a classification into 3 clusters that met relevance and statistical requirements was chosen. The cluster stability was evaluated by altering the initial sample. To determine significant differences between clusters, analysis of variance and the Kruskal-Wallis test were used for continuous variables (i.e. SCORAD and biological parameters such as serum total IgE and blood eosinophilia); Chi-2 test and Fischer's exact test were used for categorical variables. In a second step, a decision tree was constructed by using the classification and regression trees method [19]. The aim was to predict the cluster label based on a list of variables used for the cluster analysis (R package, Rpart). The trees were pruned to minimize the cross-validated error. The random forest method [20], which consists in aggregating many decision trees, was also used (R package, random forest). This method, which is based on re-sampling techniques, is more stable and can be used to determine which variables are important in the classification.

## Results

271 consecutive children consulting as outpatients for AD management were included in the study. Forty-two were lost to follow-up immediately after the inclusion visit and were not included in the analysis. Fifteen subjects had missing data for the variables used in the cluster analysis. The remaining 214 patients were included in the analysis. The baseline characteristics of the children not included in the final analysis did not differ from those of the final sample in terms of age, gender, SCORAD, parental history of asthma, food allergy and environmental factors at the time of inclusion.

### Descriptive data at inclusion

Mean age (+/- SD) was 8.51 +/- 2.79 months, 124 (57.9%) were boys, mean SCORAD (+/- SD) was 28.04 +/- 16, and 25 (11.7%) had a documented history of food allergy. Mean serum total IgE level (+/- SD) was 114.6 +/- 322.7 kU/L and mean blood eosinophilia (+/- SD) was 354 +/- 543/mm^3^. 123 (57.5%) children were sensitized to food allergens and 32 (14.9%) to aeroallergens. Results concerning the cohort are summarized in Tables 1 and 2.

**Table 1:** Clinical parameters at inclusion in the entire cohort and according to cluster analysis.

Parameters	Entire cohort n = 214	Cluster 1AD^1^ with LS^2^ n = 94	Cluster 2AD with MS^3^ n = 84	Cluster 3AD with FHA^4^ n = 36	p-value^6^
**Age (months), mean +/- SD** ^5^	8.51 +/- 2.79	8.08 +/- 2.92	8.76 +/- 2.50	9.09 +/- 2.96	0.13
**Male sex**	57.9	63.8	56	50	0.30
**SCORAD, mean +/- SD**	28.04 +/-16	25.29 +/- 14.6	32.66 +/- 16.6	24.46 +/- 15.7	**<0.01**
**Parental history of asthma**	34.1	1.1	42.9	100	**<0.001**
**Documented history of food allergy**	11.7	1.1	21.4	2.3	**<0.001**
**Cow’s milk allergy**	5.6	0	14.3	0	**<0.001**
**Multiple food allergy**	3.3	0	8.3	0	**<0.01**
**Exposure to tobacco smoke**	36	27.7	41.7	44.4	0.07
**Day care attendance**	51.9	53.2	48.8	55.6	0.75
**Socio-economic status:**					0.5
**High**	37	39.6	39.2	24.2	
**Intermediate**	37	37.4	32.9	45.5	
**Low**	26.1	23.1	27.9	30.3	
**Furred pet at home**	22	16	29.8	19.4	0.07
**Ethnic background:**					0.11
**Western Europedescent**	48	37.3	50.8	64.5	
**AfricanCaribbean descent**	23.4	29.9	24.6	12.9	
**- Other descent**	28.7	33	24.6	22.6	

All values for categorial or qualitative variables given as percentages. Boldfaced text indicates statistical significance.

^1^AD = atopic dermatitis

^2^LS = low sensitization

^3^MS = multiple sensitizations

^4^FHA = familial history of asthma.

^5^SD = standard deviation

^6^Analysis of variance and Chi-2 test when conditions allowed, Kruskal-Wallis and Fischer's exact test otherwise.

**Table 2:** Biological parameters at inclusion in the entire cohort and according to cluster analysis.

Parameters	Entire cohort	Cluster 1 AD^1^ with LS^2^	Cluster 2AD with MS^3^	Cluster 3 AD with FHA^4^	p-value^6^
	n = 214	n = 94	n = 84	n = 36	
**Total serum IgE level (kU/L), mean +/- SD** ^5^	114.6 +/- 322.7	34.8 +/- 86.6	241.8 +/- 482.9	31.3 +/- 44.3	**<0.001**
**Blood eosinophilia (cells count/mm** ^**3**^ **), mean +/- SD**	354 +/- 543	242.5 +/- 285.1	554.9 +/- 760.9	186.2 +/- 248.4	**<0.001**
**Sensitization to food allergen**	57.5	27.7	98.9	38.9	**<0.001**
**Multiple sensitizations to food allergens**	38.8	2.3	96.4	0	**<0.001**
**Sensitization to hen’s egg**	54.2	28.7	86.9	44.4	**<0.001**
**IgE level (kU/L), mean +/- SD**	6.6 +/- 15.2	1.9 +/- 5.9	14 +/- 21.4	1.5 +/- 3.6	**<0.001**
**Sensitization to cow’s milk**	49.1	5.3	72.6	8.3	**<0.001**
**IgE level (kU/L), mean +/- SD**	1.5 +/- 4.9	0.1 +/- 0.6	3.7 +/- 7.2	0.2 +/- 0.9	**<0.001**
**Sensitization to peanut**	30.8	5.3	70.2	5.6	**<0.001**
**IgE level (kU/L), mean +/- SD**	3.2 +/- 12.8	0.2 +/- 1.4	7.8 +/- 19.5	0.1 +/- 0.3	**<0.001**
**Sensitization to white fish**	1.9	0	4.8	0	**0.04**
**IgE level (kU/L), mean +/- SD**	0.8 +/- 0.7	-	0.2 +/- 0.1	-	0.2
**Sensitization to wheat**	3.7	1.1	8.3	0	**0.02**
**IgE level (kU/L), mean +/- SD**	0.2 +/- 1.8	0 +/- 0.4	0.5 +/- 2.8	-	**0.015**
**Sensitization to aeroallergens**	14.9	10.6	26.2	0	**<0.01**
**Multiple sensitizations to aeroallergens**	3.3	1.1	7.1	0	**<0.01**
**Sensitization to HDM**	3.3	3.2	3.6	2.8	0.97
**IgE level (kU/L), mean +/- SD**	0.2 +/- 1.3	0.29 +/6 1.8	0.09 +/- 0.55	0.04 +/- 0.3	0.82
**Sensitization to cat dander**	12.6	8.5	22.6	0	**<0.001**
**IgE level (kU/L), mean +/- SD**	0.8 +/- 6.9	0.1 +/- 0.4	1.9 +/- 1.1	-	**<0.001**
**Sensitization to pollens**	2.3	0	5.9	0	**0.02**
**IgE level (kU/L), mean +/- SD**	0.01 +/- 0.1	-	0 +/- 0.2	-	**<0.001**
**Sensitization to cockroaches**	0.5	0	1.2	0	0.5
**IgE level (kU/L), mean +/- SD**	0.01 +/- 0.2	-	0 +/- 0.3	-	0.46

All values for categorial or qualitative variables given as percentages. Boldfaced text indicates statistical significance.

^1^AD = atopic dermatitis

^2^LS = low sensitization

^3^MS = multiple sensitizations

^4^FHA = familial history of asthma.

^5^SD = standard deviation

^6^Analysis of variance and Chi-2 test when conditions allowed, Kruskal-Wallis and Fischer's exact test otherwise.Boldfaced text indicates statistical significance.

### AD phenotypes at the time of inclusion, based on cluster analysis

**Fig 1:**
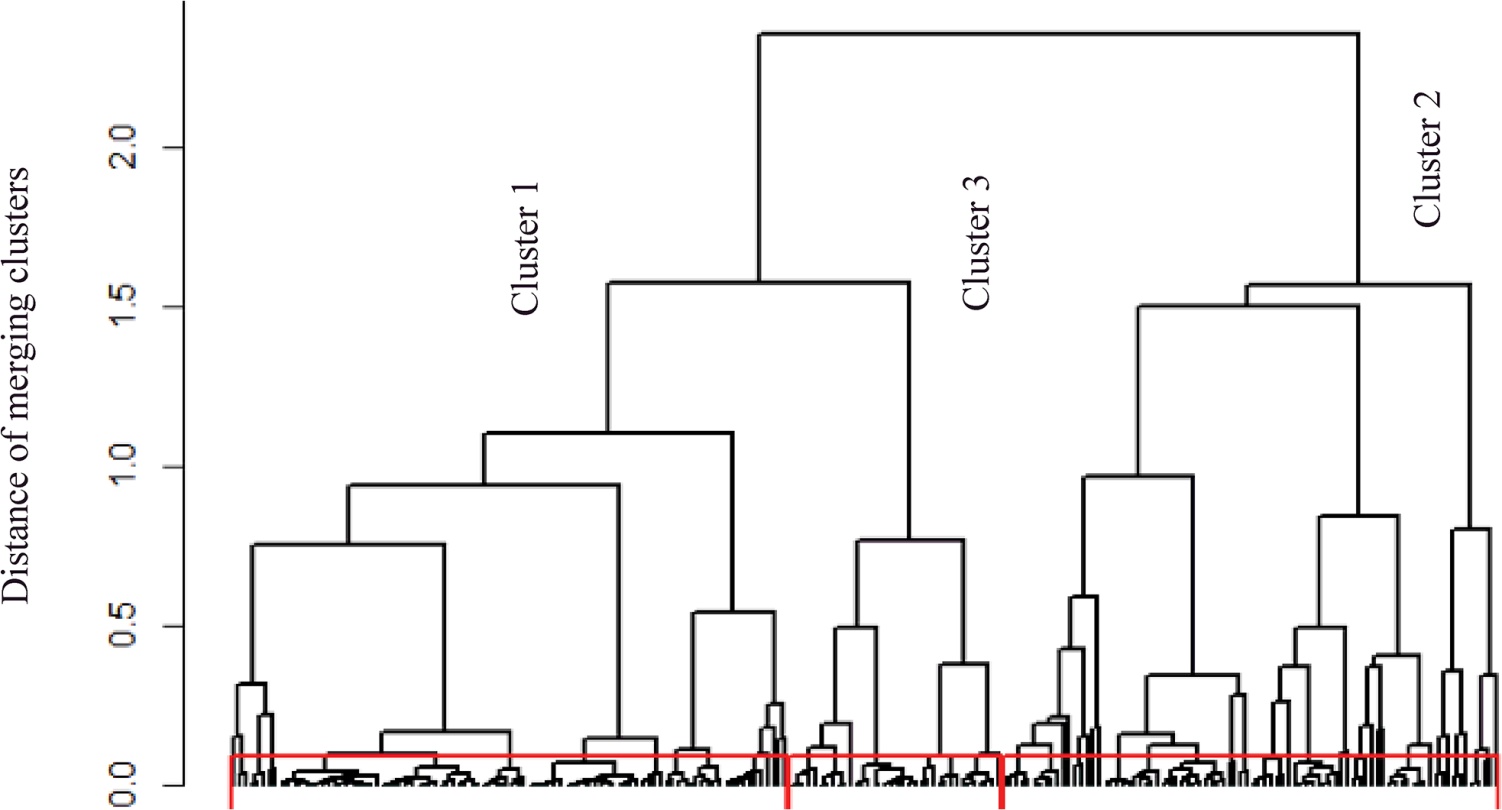
Dendogram for the entire population (n = 214), obtained with a hierarchical bottom-up clustering. Three clusters were apparent. This agglomerative approach begins with each subject as a separate cluster and merges them into successively larger clusters. By Ward’s linkage, samples were merged into larger clusters to minimize the within-cluster sum of squares. Cluster 1: “AD with low sensitization”, cluster 2: “AD with multiple sensitizations”, cluster 3: “AD with familial history of asthma”.

A hierarchical clustering approach was used to generate a classification that revealed 3 clusters of children with shared phenotypes (Fig 1). These clusters were identified using the 9 following variables: sex, history of parental asthma, severity of AD based on SCORAD, cow’s milk allergy, food allergy, serum total IgE, blood eosinophilia, sensitization to food allergens and aeroallergens.

#### Cluster 1

Cluster 1 comprised 94 children, aged (mean +/- SD) 8.08+/- 2.92 months. Sixty (63.8%) were boys. In this cluster, AD severity was moderate (mean SCORAD +/- SD 25.29 +/- 14.6) and a parental history of asthma was reported for one patient only (1.1%). Food allergy was also rare (n = 1, 1.1%). None of the children was reported to have cow’s milk allergy. Sensitization to allergens was infrequent (n = 26, 27.7% to food allergens, with 2.3% multiple food sensitizations and n = 10, 10.6% to aeroallergens with 1.1% multiple aeroallergen sensitizations). Blood eosinophilia (mean+/- SD) was 242.5+/-285.1/mm^3^ and total IgE level (mean+/-SD) was 34.8 +/- 86.6 kU/L. We called this cluster “AD with low sensitization”.

#### Cluster 2

Cluster 2 comprised 84 children, aged (mean +/- SD) 8.76+/- 2.5 months. Most of them (56%) were boys. This cluster had the highest AD severity (mean SCORAD +/- SD 32.66+/-16.6). Cow’s milk allergy was reported exclusively in this cluster (n = 12, 14.3%) and food allergy was also most frequent (n = 18, 21.4%) and frequently multiple (n = 7, 38.9%). A parental history of asthma was reported for 36 patients (42.9%). This cluster also had the highest total IgE level (mean +/- SD, 241.8+/- 482.9 kU/L) and the highest blood eosinophilia (mean +/- SD 554.9+/- 760.9/mm^3^). Sensitization to food allergens was most frequent (n = 83, 98.9%) with 81 children with multiple food sensitizations (96.4%). Sensitization to aeroallergens was more frequent in this cluster compared to the others (n = 32, 26.2%, with 7.1% multiple sensitized). We called this cluster “AD with multiple sensitizations”.

#### Cluster 3

Cluster 3 comprised 36 children, aged (mean+/- SD) 9.09+/-2.96 months. It included more girls than the other clusters (n = 18, 50%). AD severity was moderate (mean SCORAD +/- SD, 24.46+/-15.7). All children in this cluster had a parental history of asthma. Food allergy was lower than in cluster 2 (n = 1, 2.8%) and also exclusively single. None of the children had cow’s milk allergy. Sensitization to food allergen was present in 38.9% (n = 14) and was exclusively single. None of the children were sensitized to aeroallergens. Mean levels of total IgE and blood eosinophilia were the lowest in this cluster, values were (+/- SD) 31.3+/- 44.3 kU/L for IgE and 186.2+/-248.4/mm^3^ for blood eosinophilia. We called this cluster “AD with familial history of asthma”.

### Progression by the end of the follow-up

At the age of 6 years, 54 children (26.6%) had developed asthma. Forty-five of these (83.3%) had allergic asthma. No significant relationship was found between persistence of AD and asthma outcome at the age of 6. There were significantly higher percentages of children suffering from asthma at the age of 6 in clusters 2 and 3 compared to cluster 1 (respectively 36.1% and 33.3% versus 14.9%, p<0.01) and more cases of allergic asthma in these clusters (30.5 and 27.3% in clusters 2 and 3 respectively compared to 12.6% in cluster 1, p = 0.01). There was a trend towards a greater percentage of remission of AD in clusters 3 and 1, compared to cluster 2 (respectively 64.7 and 47.1% versus 40.5%, p = 0.06). The fact that the results persisted over time in spite of a slight decrease in the statistical significance of some variables attested to the stability of the cluster. Results are summarized in Table 3.

**Table 3:** Parameters at the end of the follow-up (6 years).

Outcomes	Entire cohort n = 214	Cluster 1 AD^1^ with LS^2^ n = 94	Cluster 2 AD with MS^3^ n = 84	Cluster 3 AD with FHA^4^ n = 36	p-value^5^
**AD remission**	47.5	47.1	40.5	64.7	0.06
**Asthma at the age of 6**	26.6	14.9	36.1	33.3	**<0.01**
**Allergic asthma**	21	12.6	30.5	27.3	**0.01**

All values given as percentages. Boldfaced text indicates statistical significance.

^1^AD = atopic dermatitis

^2^LS = low sensitization

^3^MS = multiple sensitizations

^4^FHA = familial history of asthma.

^5^Analysis of variance and Chi-2 test when conditions allowed, Kruskal-Wallis and Fischer's exact test otherwise.Boldfaced text indicates statistical significance.

### Classification tree and random forest

**Fig 2:**
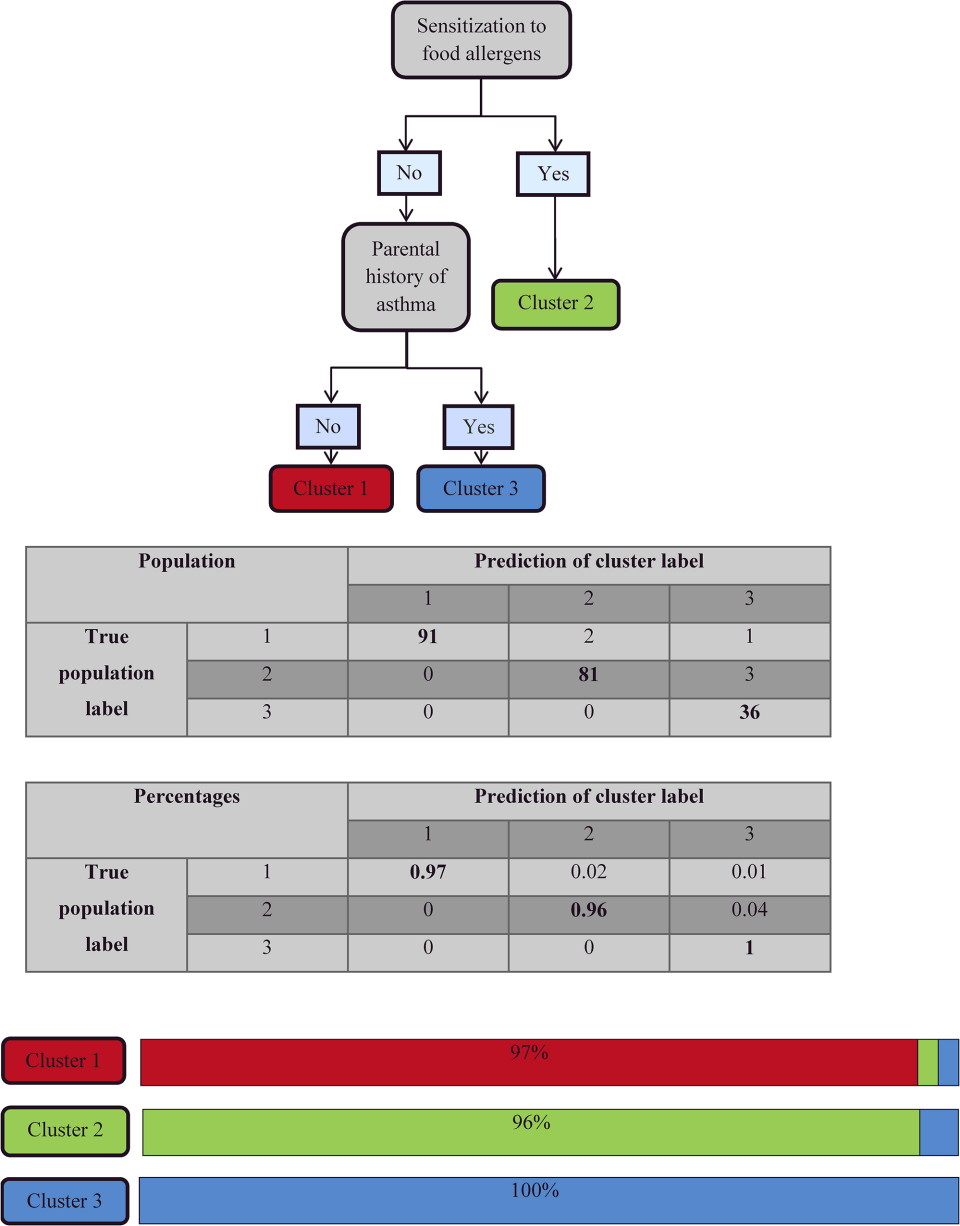
Classification tree for the entire cohort based on two variables. Each subject was assigned to one of the 3 clusters using the tree; 97% of the subjects were assigned to the appropriate cluster. Tree performance values are given in the table. Cluster 1: “AD with low sensitization”, cluster 2: “AD with multiple sensitizations”, cluster 3: “AD with familial history of asthma”.

**Fig 3:**
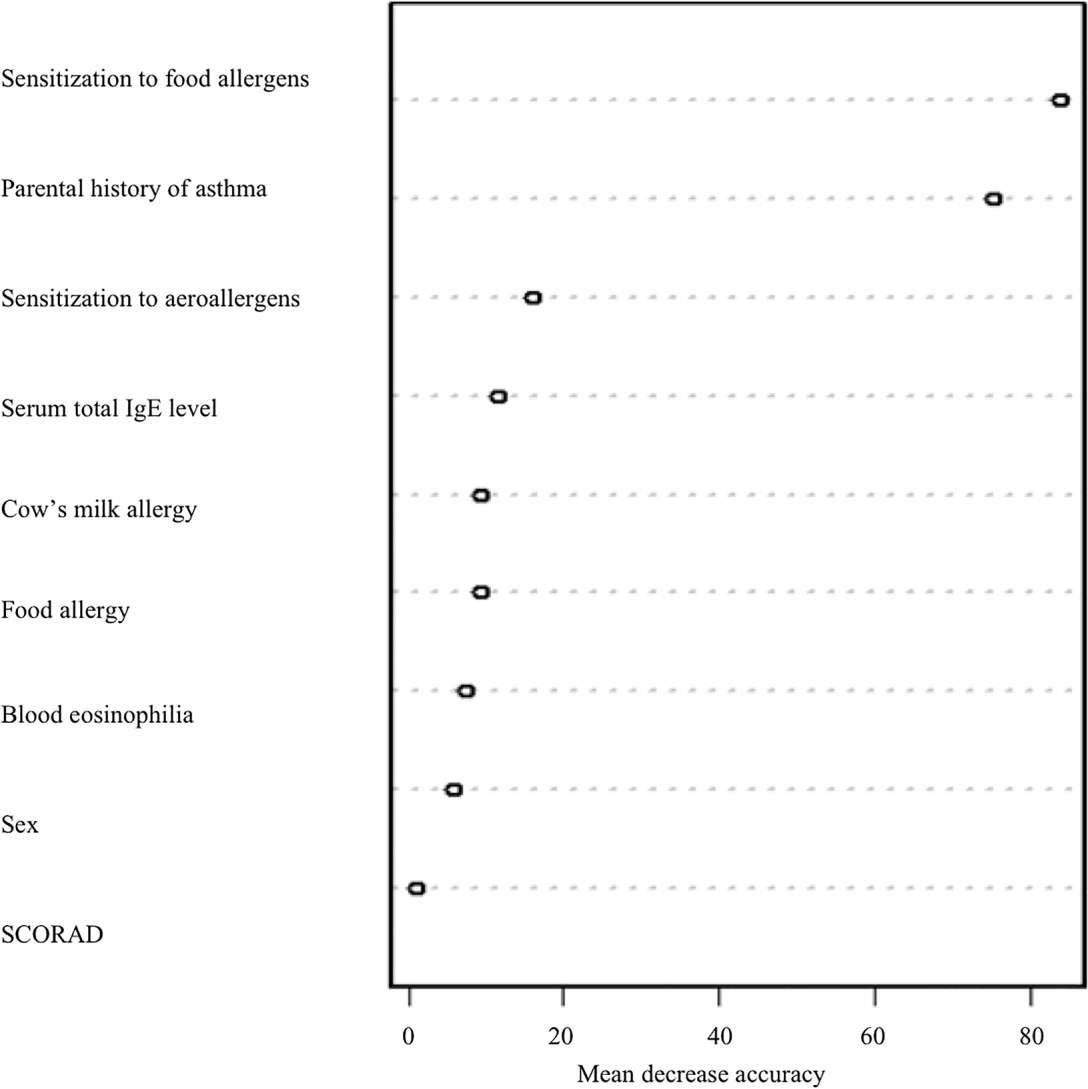
Importance measure (permutation-based mean decrease accuracy) provided by the random forest analysis. The values are not interpretable but the ranking is of interest since a high value of the importance measure is associated with a high predictive power. Sensitization is defined by specific IgE to one or more allergens ≥0.35 kUI/L. Multiple sensitizations were defined as at least two positive specific IgEs to allergens. Serum total IgE level expressed in kU/L, blood eosinophilia expressed in eosinophils/mm^3^

A classification tree using only 2 variables–sensitization to food allergens and parental history of asthma–assigned 97% of the children to the appropriate cluster (Fig 2). These two variables also had the highest random forest score of importance, supporting the relevance of this classification tree (Fig 3).

### Associations of AD phenotypes with risk factors

There was no statistically significant difference between the clusters according to day care attendance (p = 0.74), socio-economic status (p = 0.5), tobacco smoke exposure (p = 0.07), furred pet exposure (p = 0.07) or ethnic background (p = 0.24). Children in clusters 2 and 3 were more exposed to tobacco smoke compared to cluster 1 (respectively 41.7 and 44.4% versus 27.7%) and to furred pets in cluster 3 compared to clusters 1 and 2 (29.8% versus respectively 19.4% and 16%), without reaching statistical significance (p = 0.07 for both).

## Discussion

Our main finding is that we identified 3 phenotypes of early-onset AD: one phenotype mainly concerning children with low allergenic sensitization, and two with a risk of developing asthma, one for children who had a parental history of asthma and another for those with multiple sensitizations to food allergens.

### Multiple allergic sensitizations and risk of developing asthma

In high-risk cohorts of wheezing preschool children, sensitization to food allergens and aeroallergens is considered to be a risk factor for developing asthma at the age of 6 [21]. We have previously described in this cohort that multiple food sensitizations conveys a high risk of sensitization to aeroallergens at school age [2]. Furthermore, we found that the higher the sum of specific IgEs, especially IgEs to aeroallergens, the higher the risk of wheeze at school-age [21, 22]. Conversely, in this cohort with early-onset AD, a lower rate of sensitization to allergens conveyed a lower risk of subsequent asthma. Lack of eosinophilia, low level of total IgE and low level of sensitization to allergens were mostly present in cluster 1, which seems to convey a lower risk of asthma at the age of 6. This result is in accordance with previous reports of lack of eosinophilia as a predictive factor of remission of asthma in wheezing infants [16] and confirms a pattern of sensitization, especially if multiple, as a risk factor for asthma at the age of 6 [21]. The prevalence of asthma in the “AD with low sensitization” cluster (cluster 1) approached the prevalence of asthma in the general population in Western Europe [23]. Conversely, asthma prevalence was the highest in the “AD with multiple sensitizations” cluster (cluster 2) which seems to be closer to the previously described IgE-related AD phenotype [7]. Indeed, this AD phenotype is primarily characterized by high serum IgE levels, specific IgEs to food allergens or aeroallergens, and is associated with disease severity [24]. This form of AD may be considered as the initial step of the atopic march [25], as severity and early sensitization are major prognostic determinants [26, 27]. In this group of patients, immune dysregulation, including increased serum IgE sensitization to allergens and increased Th2 cytokine expression in eczematous lesions, may be the underlying pathway leading to the expression of atopic diseases such as allergic asthma [28]. To support this hypothesis, it has been suggested that co-manifestation of early wheeze and AD does not explain the increased risk of childhood asthma associated with AD, and that sensitization is the primary confounding factor in this association [9]. Gene expression seems to be different according to the phenotype, particularly in genes involved in human keratinocytes, as well as filaggrin expression which is down-regulated in IgE related AD [24]. Th2 polarization, IgE sensitization immunological pathway, and genetic background probably explain the increased risk of developing asthma in this AD phenotype, which can be seen as the atopic march model. However, reducing the pathogenesis of the atopic march to an “allergic” IgE-mediated disease as has been hypothesized may be exaggerated [8]. Thus, Pinart *et al*. have shown in a large prospective cohort study that although IgE sensitization in independently associated with excess comorbidity of AD, rhinitis and asthma, its presence accounted for only 38% of comorbidities [29]. The developmental profile of asthma is probably a far more complex mechanism, and children with early-onset AD who develop asthma later in life may belong to a particular rare genotype among the atopic diseases [30].

### Parental history as a risk factor for developing asthma in low sensitized AD phenotype

The relative weight of parental history of asthma has been a matter of debate, although it is largely accepted as a risk factor for developing asthma in general population. Thus, a personal history of AD is known to be a more predictive factor for risk of sensitization and asthma in young children than parental history of asthma in a population of children with recurrent wheezing [28]. Here, the “AD with familial history of asthma” cluster was associated with a higher risk of asthma at school-age. This would support our previous results, as a parental history of asthma has an effect on the development of childhood asthma, independently of allergic comorbidities [31]. The risk of developing asthma in this cluster may follow an IgE-sensitization independent pathway, including innate immunity defects [32]. The link between parental history of asthma, atopic dermatitis in children and the subsequent risk of developing asthma for children in this group may indicate an underlying systemic disease of impaired epithelia [33, 34]. A shared genetic association on innate immunity defects and defective barrier may explain the subsequent risk of asthma [29].

### Strengths and limitations

One strength of our study is that it was a longitudinal prospective cohort in a highly selected population of infants with early-onset AD explored annually in a standardized manner. Furthermore, all the clinical data were reported by physicians rather than by parents retrospectively. We also developed a simple classification tree with a very good performance value resulting in 97% of the children being assigned to the appropriate cluster. One limitation could be the rather small size of the cohort and the absence of a control group. We highlight the fact that, due to the relatively small sample size, environmental analyses should be taken as purely exploratory.

However, as mentioned in the introduction, we selected a rare but potentially severe phenotype in children at risk of developing asthma i.e., early-onset AD. In this context, the size of this selected population was greater than the number of patients suffering from this phenotype if selected from a large birth cohort.

## Conclusion

AD should not be seen as a single entity and as such as a systematic input leading to the atopic march. Indeed, early-onset AD corresponds to various phenotypes which do not follow the same course. Children with no parental history of asthma and no sensitization have the best respiratory prognosis. Although there is evidence that highly-sensitized children have a greater risk of asthma at school-age, atopy definition should not be limited to IgE sensitization which is insufficient to assess the risk of subsequent asthma. Thus, familial history should be another risk factor to take in account. Various pathophysiologic mechanisms leading to the clinical expression of AD associated to asthma could be associated to different genetic polymorphisms.

## Supporting Information

S1 File Dataset of ORCA Cohort (XLS)
